# Existing evidence on the effect of urban forest management in carbon solutions and avian conservation: a systematic literature map

**DOI:** 10.1186/s13750-024-00344-3

**Published:** 2024-10-03

**Authors:** Kayleigh Hutt-Taylor, Corinne G. Bassett, Riikka P. Kinnunen, Barbara Frei, Carly D. Ziter

**Affiliations:** 1 Faculty of Biology, Concordia University, 7141 Sherbrooke Avenue Ouest, Montreal, QC H4B 1R6 Canada; 2 Faculty of Forestry, University of British Columbia, 2424 Main Mall, Vancouver, BC V6T 1Z4 Canada; 3 Science and Technology Branch, Environment and Climate Change Canada, 105 Rue McGill, Montreal, QC H2Y 2E7 Canada

**Keywords:** Urban forestry, Urban tree, Conservation evidence, Literature map, Avian success, Trade-offs, Multi-objective management, Urban sustainability, Urban birds

## Abstract

**Background:**

Urgent solutions are needed in cities to mitigate twin crises of global climate change and biodiversity loss. Urban nature-based solutions (actions that protect, sustainably manage, and restore ecosystems while simultaneously providing human wellbeing and biodiversity benefits) are being advocated for as multi-functional tools capable of tackling these societal challenges. Urban forest management is a proposed nature-based solution with potential to address both climate change mitigation and biodiversity loss along with multiple other benefits. However, bodies of evidence measuring multiple outcomes (e.g., biodiversity conservation and nature-based climate solutions) remain siloed which limits conservation and management opportunities. In this article, we present a systematic map of the literature on urban forest management strategies that measure both biodiversity goals (through avian conservation) and climate change mitigation goals (through carbon storage and sequestration).

**Methods:**

Following a published protocol, we searched for evidence related to urban forest management strategies for (1) avian conservation and (2) carbon solutions within the global temperate region in academic and grey literature. In addition to Scopus, ProQuest and Web of Science Core Collection, we searched 21 specialist websites. We screened English language documents using predefined inclusion criteria on titles and abstracts, and then full texts. All qualifying literature items were coded, and metadata were extracted. No study validity appraisal was conducted. We identified knowledge clusters and gaps related to forest management strategies for both topics.

**Review findings:**

Our searches identified 19,073 articles published, of which 5445 were duplicates. The title and abstract screening removed a further 11,019 articles. After full-text screening (1762 and 1406), a total of 277 avian and 169 forest carbon literature items met the eligibility criteria and were included in the final database. We found a large knowledge base for broad-scale avian metrics: abundance, species richness. We similarly found that both avian and carbon solutions most often used broad-scale forest management components: land use type, composition, and forested area and least often considered fragmentation, connectivity, and diversity metrics (abundance, richness). The most understudied avian metrics were foraging, resources, and survival while the most understudied carbon solutions metrics were soil carbon, dead wood and organic matter and infrastructure. Avian literature most often used an experimental design (56% with comparator, 44% no comparator) while forest carbon solutions literature was dominated by observational studies (86%). In both topics, studies most often occurred over short timelines between 0 and 1 and 2–5 years. The body of evidence for both avian and carbon outcomes present a scale-mismatch between the scale of forest management strategy (e.g., land use type) and scale of application (e.g., patch). For example, the majority of studies considered forest strategies at broad scales, like land use type or composition, yet were conducted at a patch or multi-patch scale. Our systematic map also highlights that multi-city and regional urban scales are underrepresented in both carbon solutions and avian conservation and will require additional research efforts. Finally, we highlight gaps in the inclusion of recommendations in both bodies of literature. Roughly 30% of articles in each topic’s database did not include recommendations for practitioners or researchers.

**Conclusions:**

Our systematic map provides a database and identifies knowledge gaps and clusters of urban forest management strategies for (1) avian conservation and (2) carbon solutions. Overall, our map will allow researchers to fill existing gaps in literature through new research investigations, meta-analyses or systematic reviews while also pointing policymakers toward strong knowledge bases in addition to understudied or mismatched areas that require more funding.

**Graphical Abstract:**

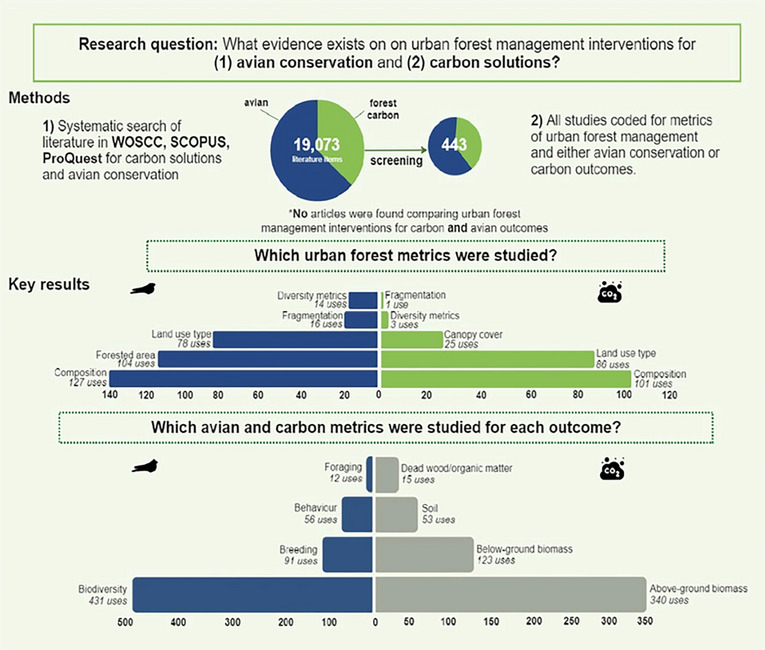

**Supplementary Information:**

The online version contains supplementary material available at 10.1186/s13750-024-00344-3.

## Background

Urgent solutions are needed to mitigate global climate change and biodiversity crises which threaten the ecosystems that support human wellbeing. This is particularly true in cities, where the majority of people live globally [1]. Because of the complex and widespread nature of these challenges, leading scientific and environmental policy bodies have concluded that broad, systemic actions are needed from individual to international scales, in almost every aspect of human life [2]. Expanding urban areas are increasingly feeling the impacts of climate change and biodiversity loss but are also at the forefront of delivering solutions in these twin crises [3]. Urban nature-based solutions, actions that protect, sustainably manage, and restore ecosystems while simultaneously providing human wellbeing and biodiversity benefits [4], are advocated for as multi-functional tools capable of tackling societal challenges [5]. “Nature-based solutions” has emerged as a powerful umbrella term and is currently widely used in policy and programming around the world (e.g., Natural Climate Solutions Fund in Canada, Department of Interior Nature-based Solution Roadmap in the United States). Importantly, the concept of NbS encourages strategies and interventions which are integrated and provide multiple benefits – as opposed to solutions focused on single outcomes. For example, managing forests for carbon sequestration and wildlife habitat also supports myriad other co-benefits, including mitigation of stormwater flooding and extreme heat and supporting the mental and physical wellbeing of urban residents [6, 7]. Such urban nature-based solutions include the conservation, management, and restoration of urban ecosystems from forests to wetlands, and encompass many interventions such as trees, rain gardens, bioswales, and more [8].

Among examples of nature-based solutions, protection, management, and restoration of urban forests have been identified as crucial strategies for both mitigating climate change and supporting biodiversity [8]. Urban forests can be defined as social-ecological systems of trees, associated vegetation, and people within urban areas [9]. Research on urban forests is multi- and interdisciplinary, from fields such as forestry, arboriculture, ecology, planning, and social sciences [8]. The diversity of disciplines engaged in research on urban forests can be thought of as a strength, leading to new and needed insights, though there is also evidence that bodies of literature on urban forests are evolving in ways that do not intersect [10] and many unexplored avenues of research still remain. Literature on the ecosystem services of urban forests can be especially susceptible to a lack of integration, as expertise on specific ecosystem services can be found in separate disciplines, such as biology for habitat provision, social sciences for mental health benefits of nature, and hydrology for stormwater management, despite being linked by the same underlying system [11]. In urban forests, there can also be divisions and challenges as a result of scale of inquiry—such as between research specialising in the scale of individual trees vs whole cities—and subsequent differences in recommendations [12]. Meanwhile, for nature-based solutions, such as urban forest management, to be successfully implemented in urban areas, practitioners and policymakers require that these diverse threads of literature be synthesised into evidence-based recommendations to support decision-making for strategic sustainability goals [13, 14]. Additionally, further information is needed on the gaps in knowledge that remain to support future research efforts to better determine the effectiveness of these strategies.

Urban forests have a high potential to provide solutions to the twin crises of climate change and biodiversity loss. There is evidence showing urban forests' capacity to sequester and store carbon in service of climate change mitigation [15, 16]. This body of research has been mobilised in national-scale governmental initiatives, for example, in Canada’s Natural Climate Solutions Fund which highlights urban tree planting as a key nature-based climate solution. While the global overlap between urban growth and biodiversity hotspots is most evident in the tropics, similar trends are occurring in temperate regions [17]. For example, Canada’s priority conservation areas are disproportionately located in the southern parts of the country where urban development is also concentrated. This results in less than 5% of Canadian land providing habitat for over 60% of species at risk, meaning that urban forests often provide key habitat to support biodiversity, particularly birds [18]. Given that urban forests can support high numbers of bird species and are crucial stopover sites during migration [19–21], management efforts that focus on avian success are both highly relevant and tangible for urban management [22, 23]. However, many knowledge gaps remain, moreover, synthesis of the existing evidence for biodiversity and carbon solutions has not been examined.There is also growing interest amongst urban foresters and arborists to tailor management decisions to integrate wildlife knowledge [24, 25].

However, despite the evidence on the benefits of urban forests, many cities find themselves growing “greyer not greener” [26, 27]. To date, bodies of evidence related to urban nature-based solutions remain siloed, which in turn limits the management and conservation efforts being implemented. For example, urban forest management strategies tend to focus on a single aspect of “conservation” (e.g., avian diversity or climate regulation) despite having high capacity to address both climate change mitigation and biodiversity along with multiple other benefits [8]. Rigorous and translatable research on the trade-offs and synergies of different management strategies is needed to support the decision-making of policymakers and practitioners and to ensure best outcomes.

**Fig. 1:**
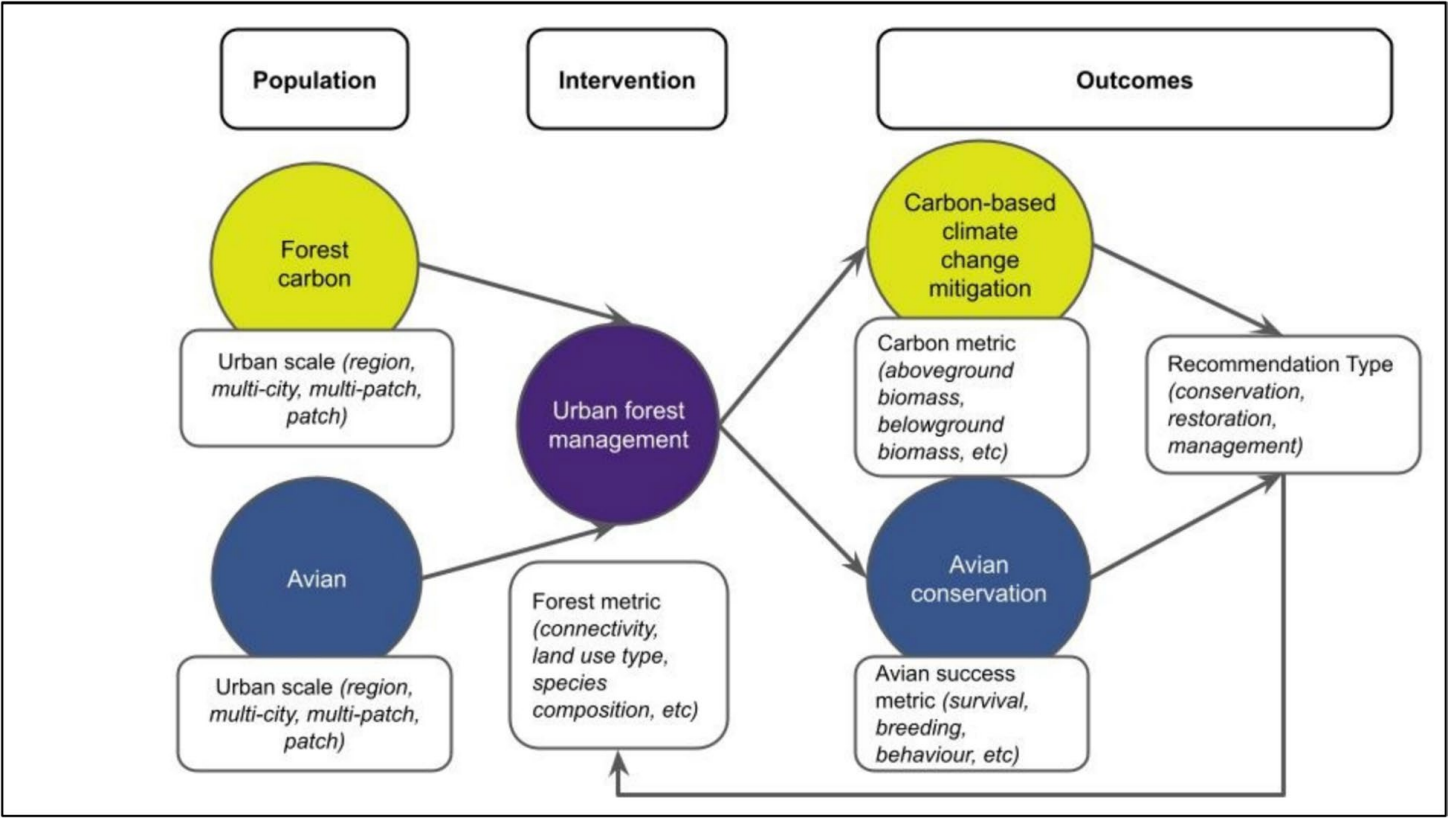
Theory of change linking our two bodies of literature, (1) avian and (2) forest carbon, through urban forest management (forest metrics) towards the outcomes of avian conservation or carbon solutions. Arrows visualize the flow of information towards two desired outcomes which then inform recommendations for further intervention. Variables extracted in the full-text analysis are denoted in white boxes

In this article, we present a systematic map of the literature on urban forest management strategies for climate change mitigation, specifically carbon solutions (e.g. carbon storage and sequestration), and to support biodiversity, specifically birds and/or species at risk. Thus, we present a theory of change linking urban forest management interventions with outcomes for avian conservation and carbon solutions (Fig. 1). This theory of change shows a two-pronged approach is necessary to synthesise research across disciplinary boundaries, in this case, forest carbon studies and avian studies. To our knowledge, such an approach, as documented in our published systematic map protocol [28], has not been undertaken to systematically review literature on these bodies of research.

### Stakeholder engagement

A key component of our effort to assess and synthesise the evidence on urban forest management for avian conservation and carbon solutions has been to (1) establish a team of researchers from multiple disciplines, (2) conduct an open call for literature (see Additional file 1), including reaching out to relevant organisations, and (3) create a public-facing database for community members, practitioners and researchers to engage with the evidence compiled in our systematic map [29]. Producing our systematic map is also part of the broader project “The Birds and the Trees” which aims to co-create strategies to build cities which nurture both biodiversity and people, whether it be through conservation, management, or restoration. The team developed and built the project, search strings, as well as ROSES elements meeting on a bi-weekly to monthly basis. The team followed all the Collaboration for Environmental Evidence (CEE) methodological steps for systematic maps.

### Objective of the review

The goal of our work was to conduct a systematic map of two existing bodies of literature to guide effective urban forest management to maximize multiple benefits to climate change and biodiversity crises (species at risk not included in final dataset due to lack of evidence) and mobilize the results through a public facing website and stakeholder engagement. Here, we map the existing primary evidence on best practices for urban forest management strategies for (1) avian conservation and (2) carbon solutions in temperate regions. The principal research questions were:

1. What evidence exists on the effects of urban forest management strategies to support avian and/ or species at risk conservation?
2. What evidence exists on the effects of urban forest management strategies for carbon solutions?

In addition to our primary research questions, we will also address the following sub-questions:

1. What are the main themes in urban ecological research that have addressed urban forest management strategies to support avian and/or species at risk conservation?
2. What are the main themes in urban ecological research that have addressed urban forest management for climate-solutions (e.g., carbon storage and sequestration)?
3. What are the current trends and research efforts? Are there evidence clusters or knowledge gaps with potential for generating new knowledge?
4. Are there opportunities for multiple benefits across climate mitigation and species (avian or species at risk) conservation through urban forest management?

Addressing these questions will advance our understanding of urban forest management strategies applicable to two crucial conservation and climate change mitigation goals. Our map will highlight the gaps in the literature, including countries that are conducting most research and subject areas where further investigation is needed. This will inform future research directions and aid policymakers in moving forward with urban nature-based solutions work.

## Methods

### Deviations from the protocol

The protocol [28] was followed as closely as possible. The wording of our objective has been updated since the published protocol to include more succinct and consistent terms (e.g. “carbon solutions” instead of “carbon climate mitigation”). We originally sought to include both birds and species at risk (avian or other) in the avian search, however there was a strong bias in the literature towards bird studies, and we failed to identify any relevant literature for species at risk conservation through urban forest management strategies in temperate areas. While this bias may indicate a lack of specific literature, there is a gap in our evidence synthesis regarding forest management strategies for species at risk conservation in urban areas. Some categories of metadata were added or refined to include some new information (additional columns) in the evidence base (see Additional File 2). Finally, we plan to report some supplemental results from our database that were out of scope for our research objectives yet include potentially useful information for stakeholders in urban forestry (Additional File 2) on an online, public data repository (Zenodo).

### Search for articles

#### Search string

We composed our search string in accordance with the key components of the question representing Population, Exposure, Comparator, and Outcomes as outlined in the protocol [28]. The search string was used for Web of Science Core Collection (using “topic” search mode) and SCOPUS (using title, keywords, and abstract mode). In both search strings, the dollar sign ($) was used to accept single or no added characters, useful when searching to retrieve plural and singular forms. Quotation marks were used to search the exact word order.

Upon the recommendation of Concordia University’s biology subject-matter librarian, a simplified search string was tested and constructed in ProQuest to specifically target theses and dissertations that may not be captured through peer-reviewed journals. The search was used to obtain the best comprehensiveness and efficiency to target each subject. The search terms were composed of keywords targeting the main terms for each topic (see Additional File 3).

Search strings were tested and constructed in the Web of Science Core Collection and SCOPUS to obtain the highest efficiency and best comprehensiveness as noted in the protocol [28] (Additional File 3). All searches were performed using English terms and all relevant international literature published in English was included in this systematic map, including diverse bibliographic documents (e.g., journal articles, theses, book chapters and technical reports). We worked closely with a biology subject-matter librarian throughout this process to ensure appropriate search strategies were followed.

#### Search sources

Publication databases and organisation websites were searched without any time restriction. All searches were undertaken between April and September 2022. We conducted an open call for evidence for grey literature through social media and through relevant networks of colleagues with expertise from October to November of 2022 (see Additional File 1).

#### Bibliographic databases

Title, abstract and keywords restrictions were used in the SCOPUS and Web of Science Core Collection databases, while the ProQuest search was restricted to abstract only. All databases were accessed with a subscription of Concordia University.

#### Grey literature search

A total of 20 specialist organisation websites were searched for each topic (Tables 1 and 2, Additional File 1) to collect technical reports, guidelines, or management plans related to our research question. For each organisational website, we used the shiny app “greylitsearcher” developed by Haddaway [30] using a targeted search string for each topic as presented in the methodological protocol (see Additional File 3).

**Table 1:** Article inclusion and exclusion criteria for the ***avian group***, summarised from the manuscript. The use of italics indicates changes that were made after the protocol [28] was published.

Screening criteria	Included	Excluded
Population	Any bird species and/or species at risk in the North (23.5°N to 66.5°N) or South (23.5°S to 66.5°S) temperate regions. Bird species can be residents (e.g., non-migratory) or migratory at any life stage. Exotic species and non-natives that have been naturalized to North America will be included (e.g., European starling). Only studies located in urban ecosystems are considered.	Studies that evaluated the effects of urban forest management on avian or species at risk in rural areas. ***If it is unclear whether the area is urban, we will use the UN Statistical Commissions international threshold to include cities, towns, and semi-dense areas with a population of at least 5000 inhabitants and a density of at least 300 inhabitants per km². If information cannot be sourced, it will be excluded.***
Intervention	Studies that evaluated the effect of urban forest management strategies (e.g., habitat protection, tree planting, composition, structure, reforestation, diversity) as it relates to avian or species at risk. Relevant causes of change include urbanization, habitat loss, fragmentation, human-caused green space interventions, tree management, urban forestry related strategies (restoration, conservation* (e.g., habitat area or configuration)*, regeneration, protection *(maintaining forest buffer zones)*). Forest component must be a tree or woody species with DBH > 5cm.	*Not sufficient for work to be conducted in urban forest or its related components. Research must relate trees or urban forest management and its characteristics to bird success directly (e.g., insecticide application to fruit trees, tree species composition, urban forest area).* *Forest components indirectly related to trees such as: lawns, non-tree related vegetation (e.g., perennial plants, annuals, soil, grasses etc.)* *Studies that include urban pixels or land-use types, however, don’t reference or define urban forest components. * *Roadways connecting urban areas.*
Outcome	The reported measured outcome should indicate some change in avian or species at risk success, broadly defined to include any measurement related to a change in avian/bird or species at risk at one (or all) three levels: (1) community (richness, diversity, relative abundance, species presence and absence) (2) population (trends, patterns, abundance), and (3) individual (fecundity, survival and mortality, performance). Only studies that evaluated a direct response (outcome) of some aspect of avian or species at risk success listed above.	Studies that only evaluated an indirect response to altered urban forest management. For example, authors make an indirect link between the measured outcome of urban forest management and its “potential” impact on avian or species art risk. *Social behaviour (E.g., associations, social stability).*
Comparator	Relevant comparators included: (1) urban and rural, (2) separate but similar green space types with no intervention, (3) an alternative level of urban forest interventions on the same or different study green space. Studies that look at trends without true comparators: temporal trends of species success (urbanization), or spatial trends that do not include “control” sites.	
Language	English as full text	Any study that is not in English at full text

**Table 2:** Article inclusion and exclusion criteria for the ***carbon group***, summarised from published protocol [28]. The use of italics indicates changes that were made after the protocol [28] was published.

Screening criteria	Included	Excluded
Population	Any component of the urban forest (trees, shrubs, woody species) in North or South temperate regions or land use types that contain urban forest components in public and private domains: vacant lots, parks, streets, private yards, cemeteries, institutions, and commercial spaces.	Studies that evaluated only the vegetative component of the urban forest (e.g., tree species, canopy). Studies that measure forest soil with no link to vegetative components.
Intervention	Studies that evaluated the effect of urban forest management as a climate solution for carbon storage and sequestration. Relevant strategies broadly include tree species selection and planting, tree crown area, protected area, green space management, connectivity, and composition.	*Studies focused on land-use change and carbon capacity (e.g. how carbon storage is altered as land-use changes). Studies that only quantify carbon storage or sequestration in monetary values ($ dollars).*
Outcome	The reported measured outcome should indicate or measure differences in carbon storage or sequestration for improved climate mitigation. Broadly defined to include any measurement related to carbon stored, rates of carbon sequestration in urban areas.	Studies that indirectly link urban forest characteristics to carbon storage and sequestration. For example, studies that infer potential changes in carbon storage and sequestration without any direct measurements.
Comparator	Relevant comparators included: (1) urban and rural, (2) separate but similar green space types with no intervention, (3) an alternative level of urban forest interventions on the same or different study green space. Studies that look at trends without true comparators: temporal trends of species success (urbanization), or spatial trends that do not include “control” sites.	
Language	English as full text	Any study that is not in English at full text

When searching for evidence hosted on specialist organisation websites, websites in the avian component were searched with the keywords “urban forest bird” and websites in the forest carbon component were searched with the keywords “urban forest climate change.” In cases where these keywords did not yield relevant results, the websites were searched with the keyword “urban forest.” A maximum of ten references were downloaded from each organisational website to ensure research team members could effectively screen all sources.

#### Estimating the comprehensiveness of the search

During the scoping process, the search terms were tested using Web of Science (WOS) and SCOPUS. The review team compiled a list of 10 articles that are considered as important and highly relevant to each group of literature (avian or forest carbon) thus, a total of 20 benchmark papers (listed in Additional File 2). Each respective search string was modified and refined until all benchmark publications were retrieved. With all the results extracted (WOS, SCOPUS), 10 out of 10 articles were retrieved for each topic respectively, indicating 100% comprehensiveness according to benchmark articles. For the forest carbon group, two articles, Nowak et al. (2002) and Vieira et al. (2018), were not captured in the WOS collection, however, they were included in the SCOPUS collection. All articles were captured in both WOS and SCOPUS for the avian group. The search string at 100% for ten benchmark articles for each topic was considered satisfactory to move forward. Final search strings were conducted April 4th, 2022.

#### Assembling and managing search results

Once record extraction from each database and website was completed, we reassembled records from all sources into csv files and uploaded them to Rayyan [31]. Records from SCOPUS, Web of Science and ProQuest were exported from Zotero and uploaded to Rayyan to merge records from different database sources. Search results from the grey literature were manually uploaded to Rayyan using pdf, excel, or csv files. We removed all clear and partial duplicates using the duplicate detection tool in Rayyan.

### Article screening and study eligibility criteria

#### Screening process

We followed a two-stage filtering process and a pre-defined screening and study eligibility criteria [29] using Rayyan [31]. Titles and abstracts were screened during the first stage, followed by full texts.

Full texts were retrieved for all selected abstracts using journal access and subscriptions via Concordia University. If the articles were not retrievable, they were labelled as inaccessible. Open-source versions of articles were retrieved whenever possible. If not found, unretrievable full texts of accepted abstracts were not screened (see Additional File 4). Any titles and abstracts that did not meet the inclusion/exclusion criteria (see details in the eligibility criteria section) were excluded in the full-text screening stage. No screened articles were authored or co-authored by the screeners.

To ensure consistency in the screening process, Cohen’s kappa coefficient [32] was calculated on a list of articles screened independently by two screeners. Before the coefficient was run, a screener was trained. The screeners met to practice, discuss, and adapt the eligibility criteria on 50 titles and abstracts of each topic followed by the 10 accepted full texts. The goal of these meetings was to confirm the understanding of the eligibility criteria. Cohen’s kappa coefficient for the title and abstract screening stage was 0.75. Finally, on the full texts retrieved, Cohen’s kappa coefficient was 0.75 (avian) and 0.8 (forest carbon). At each screening stage, the reviewers met to discuss all remaining discrepancies.

#### Eligibility criteria

Articles were deemed eligible for inclusion using the inclusion and exclusion criteria presented in Tables 1 and 2. The inclusion/exclusion decisions were reported at both title and abstract and full-text screening stages. We followed the guideline recommendations by including reasons for exclusion that were also reported during the full-text screening (see Additional File 4).

During the title and abstract screening, screeners followed a list of questions (decision tree) for each respective topic (avian and forest carbon) related to population and outcome (see Additional File 1). An article's title and abstract had to meet all the inclusion criteria questions to be included. If an article met the inclusion criteria for the population questions but not the outcome, it was excluded. Since we were targeting primary studies only, we did not consider documents of methods, reviews or any policy analysis that did not report either avian conservation or carbon-based climate mitigation data.

The full-text screening stage assessed whether or not an article was to be included in the final dataset. Whole articles were assessed in detail using the inclusion/exclusion criteria for the respective component (Tables 1 and 2) and considered for inclusion.

#### Study validity assessment

The validity of evidence was not assessed in this systematic map, but information was coded related to elements that may provide very preliminary indications of internal validity such as study design, presence of a comparator, and timescale. This information is not intended to provide a comprehensive assessment of study quality, but to highlight details on different study types.

#### Data coding strategy

The metadata from all included articles, for both topics were coded in a data extraction file. The metadata is detailed in four codebook sheets in the Additional file 2. For each article of the avian and carbon search, we extracted information on (1) bibliographic information, (2) population and intervention type (4) spatial scale of study, (5) location of study, (6) study design (comparator used), (7) main result, and (8) recommendations for intervention (Table 3).

**Table 3:** Categories and definitions for extracted components of included literature in **avian group and forest carbon** group. If methodology referenced methods in another paper/journal article, readers found the article referenced and sought out additional details of methodology from the paper mentioned

Domain	Category	Definition
Bibliometrics		
Article ID Citation Authors Country of First Author Study Country Publication Year		Unique identifier for article Article citation in style of environmental evidence List of all authors Country linked to article’s first author Country where research was conducted What year was the article published
Descriptive Metrics		
City Latitude/Longitude Hemisphere Data collection year (start/end) Scale of measurement Comparator used?		What city (if listed) did the study take place in Coordinates in degrees, minutes, seconds Northern or Southern What year did data collection begin and end? Individual, population or community Was a comparator used? (experimental design)
Urban scale	Patch Multi-patch Region Multi-city	Considers local-scale differences from a single patch type in an urban context (e.g., park) Considers local-scale differences from multiple patch types in an urban context (e.g., park and vacant lots, urban rural gradient) Considers multiple urban areas within the same geographical area Considers multiple city landscapes in different geographical regions
Forest Carbon (Population)		
Carbon Metric	Aboveground biomass Belowground biomass Infrastructure Soil Dead wood/organic matter	Carbon measured by calculations considering aboveground forest components (allometric equations, etc.) Carbon measured by calculations of forest components belowground (roots, shoots etc.) Carbon stored within infrastructure (buildings, benches, materials) Carbon stored in urban forest soil Carbon stored in dead wood and organic matter within the urban forest (logs, leaf litter etc.)
Avian (Population)		
Avian Common Avian Latin		Common name(s) of bird species (listed only if 10 or less) Latin name(s) of bird species (listed only if 10 or less)
Survival	Individual survival/mortality Nesting survival	The rate of mortality within a population or community The rate of survival of eggs and nestlings within a population or community
Diversity	Diversity metric Abundance	Considers a metric of diversity (e.g. genetic, species, functional) Considers the abundance of one or several bird species
Breeding	Macro Meso Micro	Considers the effect of rural to urban landscape on breeding success Considers the effect of landscape structure (configuration, composition etc.) on breeding success Considers the effect of local habitat variables on breeding success (e.g., parasitism, predation, nest abandonment, nest structure, flight initiation distance FID)
Behaviour	Foraging Oral communication Migration	Describes foraging behaviour Describes birds’ ability to communicate through song Considers birds migration patterns, distances, and success
Demographics/ Patterns	Population changes Community changes Species distribution	Estimates trends in population metrics Estimates trends in community metrics Models the estimated distribution of species across a landscape
Resources	Intra-specific competition Inter-specific competition	Describes competition between species Describes competition among species
Forest (Intervention)		
Forest Metric	Land use type Forested area Fragmentation Connectivity Canopy Cover	Assesses types of green space containing trees within the urban matrix (e.g., parks, residential land, institutional, commercial, woodland, street trees etc.) Considers the amount of forested area (land area with forest) Considers the degree of fragmentation of forest habitat Considers the connectivity of trees or components of the urban forest within the urban matrix Considers the amount of canopy cover
	Composition Diversity metric Native species Exotic/invasive species Individual tree management	Considers the composition of tree and shrub species, age or size or structure Considers a metric of diversity (e.g. richness, Shannon diversity, functional diversity) of forest habitat Considers the quantity of native and/or exotic species Considers the quantity of invasive tree or shrub species Considers changes to individual tree maintenance and/or management (e.g., insecticide-use, fungicide use, pruning etc.)
Outcomes		
Main Result Recommendation Type Recommendations		Text summary of the main result related to either (1) avian conservation or (2) carbon solution Conservation, restoration and/or management Text summary of recommendations

The coding was undertaken in three steps. First, coding was tested on five articles and was undertaken by three reviewers (KHT, CC, RK) for the avian group and three reviewers (KHT, EC, EP) for the forest carbon group during a virtual meeting. This initial meeting ensured that each reviewer understood the metadata and allowed the team to refine the categories of extraction when necessary.

Second, reviewers (carbon: KHT, DMR, avian: KHT, RK, CC), each separately coded a test sample of 10 articles, and compared their extracted data interpretations. Differences were discussed and new adjustments were made as needed. In some cases, additional detail was added to inclusion criteria (see italics Tables 1 and 2). Differences occurred, for example, in how to code metadata (e.g. title or detail), or how to deal with ambiguous articles. Once all consistency exercises were passed, the articles were divided equally between reviewers and each reviewer reviewed the articles assigned to them individually.

Lastly, the avian team (KHT, RK and CC) coded a total of 277 articles and the forest carbon team (KHT, DMR, ER, and EC) a total of 169 articles, cross-checking specific articles identified as difficult to code. For the grey literature, two reviewers (CB and KHT) conducted comparison coding of one document (a doctoral thesis), after which CB coded 16 forest carbon documents and KHT coded one forest carbon and two avian documents. During all data extraction, we avoided any interpretation of information from articles and concentrated on extracting raw information or direct quotes when possible. Continuous communication and discussion between coders throughout the process, especially with the lead coder KHT, ensured a common understanding and application of the criteria.

Many individual articles included more than one avian success metric or forest management intervention (for example, breeding and composition, or land use type and individual tree management). These were coded as multiple-response variables, allowing a study to be more accurately portrayed. Because articles were allowed to be coded with multiple responses for a single variable, the counts of articles within certain categories may sum to more than the total number of articles in the database. When information was not present in an article related to the variable being assessed, coders entered “NA.”

#### Data mapping method

The database was managed in Microsoft Excel and analysed using R version 4.3.0 [33]. All mapping (descriptive statistics and figures) was completed using the ggplot2 package in R [33]. The final database was compiled into one file available at Additional File 2. See https://github.com/Kayleighht/Syst_Map_Avian_Forest for full scripts and Metadata.

Knowledge clusters and gaps were identified by comparing frequencies of articles across the variables common to both groups (e.g. urban scale, land use type) and the outcome variables for each group (e.g., avian metrics and carbon metrics). Bar charts and heat maps were created to present results on current knowledge, hot spots, and research approaches on each respective topic.

### Review findings

#### Review descriptive statistics

**Fig. 2:**
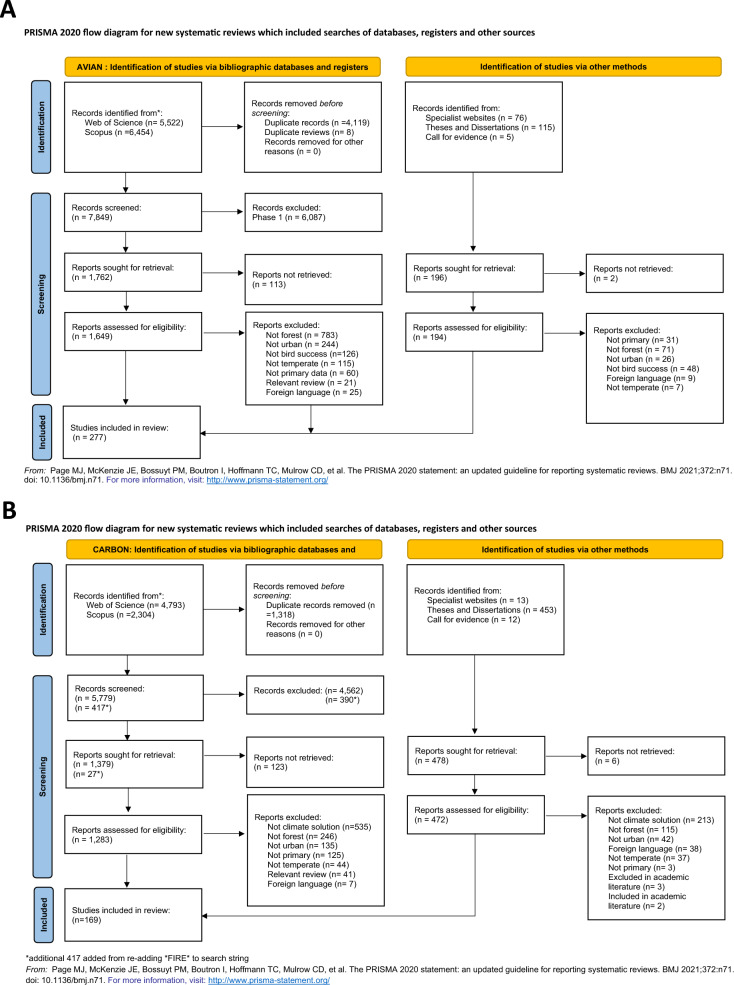
PRISMA chart for (**A**) avian group and (**B**) forest carbon literature following ROSES guidelines

The literature searches retrieved 10,315 peer-reviewed articles from Web of Science and 8758 articles from Scopus. We identified 11,976 articles for the avian component and 7097 articles for the forest carbon component (see Fig. 2ab). Forty-one forest carbon and 21 avian review articles were identified in the search, the citations of which were searched among the initial search results, and new articles were added if considered relevant to our research questions. After removing duplicates (avian, n = 4127, forest carbon, n = 1318), the title and abstracts of 7849 avian articles and 6196 forest carbon articles were screened. Full texts were retrieved for 1649 avian articles and 1283 forest carbon articles (out of which 113 avian articles and 123 forest carbon could not be retrieved) and assessed for eligibility. A total of 429 journal articles (avian, n = 275, forest carbon, n = 150) were included in the final systematic map.

The grey literature search resulted in additional studies for both the avian and forest carbon groups from specialist websites (n = 89), ProQuest Dissertations (n = 568), and a Call for Evidence (n = 17). Seventeen forest carbon and two avian grey literature articles were deemed eligible for inclusion and integrated into the final dataset, resulting in a total of 446 articles (forest carbon, n = 169, avian, n = 277). The full reference list of eligible articles can be found in Additional File 2, and the list of excluded full-text articles with the primary reason for exclusion can be found in Additional File 4.

#### Publication type and publisher

**Fig. 3:**
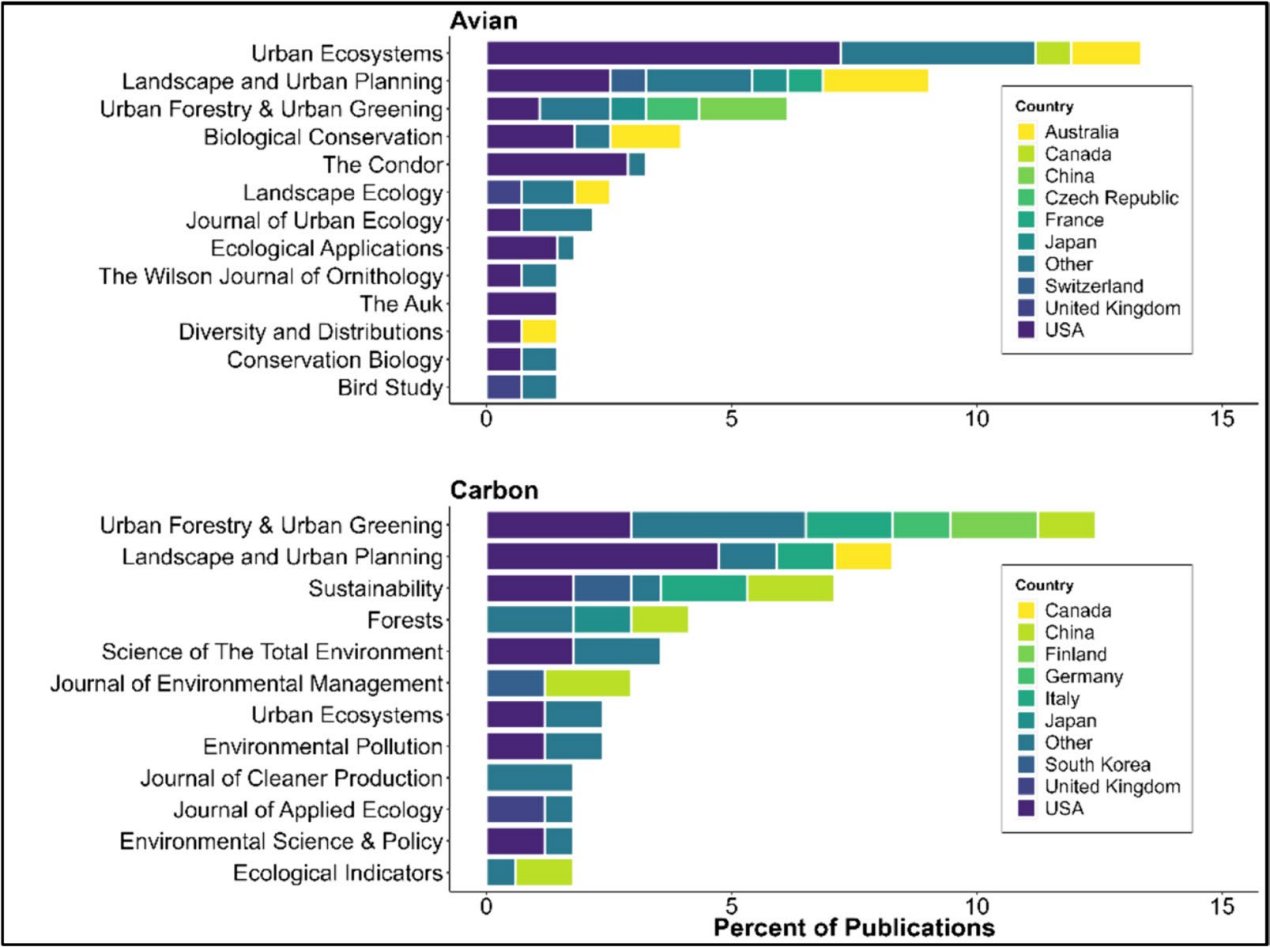
Percent of publications according to country of first author and journal for avian and forest carbon groups of literature

The vast majority of the studies included were scientific journal articles (n = 428), comprising 95% of the total dataset, followed by theses (n = 17), and book chapters (n = 2). There was one article each of the following types: symposium article, government report, NGO report. Articles across both topics were published in a total of 181 (108 and 73) different journals, the most popular being *Urban Forestry and Urban Greening* for forest carbon literature and *Urban Ecosystems* for avian literature (Fig. 3).

#### Year and location of publication

**Fig. 4:**
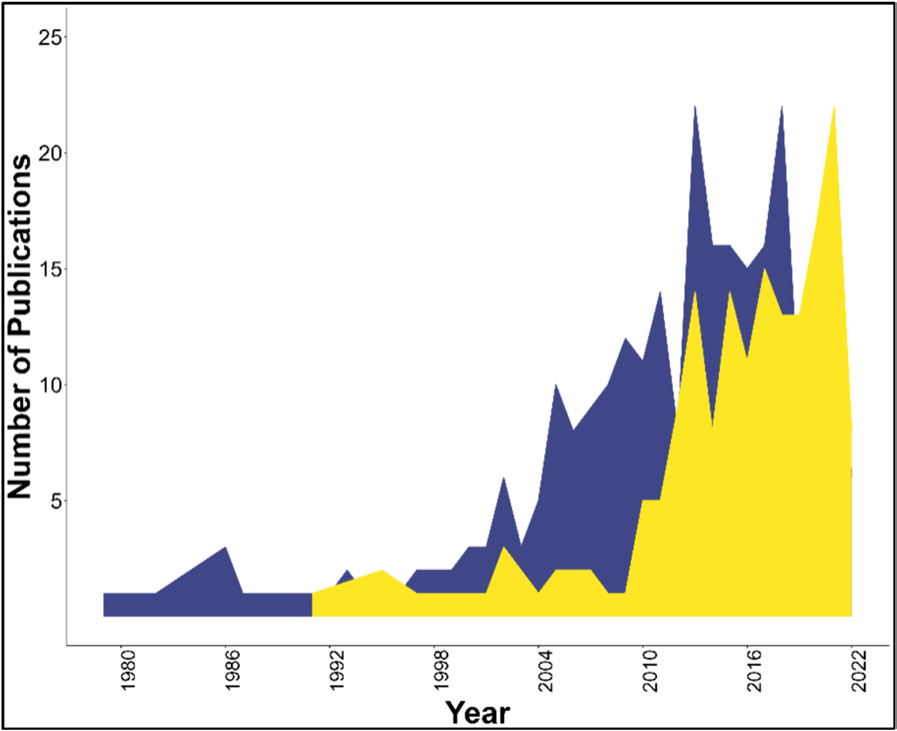
Temporal trend showing the number of articles published per year, for avian (blue) and forest carbon (yellow) groups of literature. Note that articles for 2022 include articles until April 2022 and does not include all articles of the entire calendar year

**Fig. 5:**
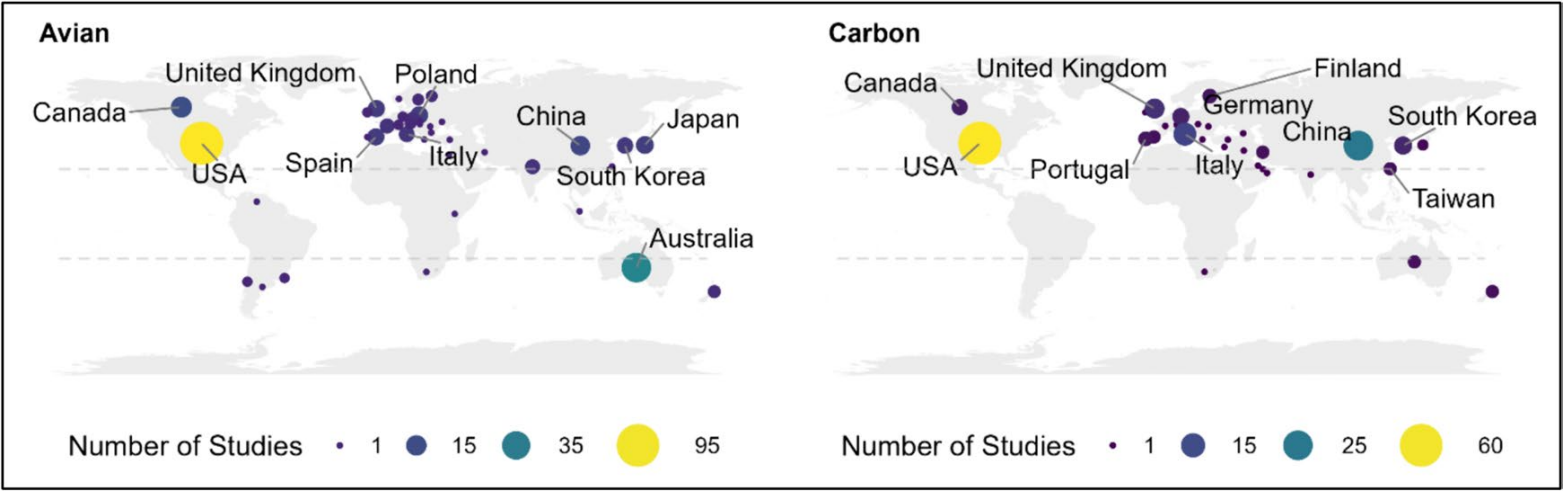
Spatial distribution of articles according to country of first author included in our dataset for avian and carbon groups. Note, our systematic map was limited to studies conducted in temperate regions (outside the dashed grey lines)

The final dataset covered a period from 1979 to 2022 (Fig. 4), with the avian articles beginning in publication 12 years earlier than the first forest carbon article among the 429 total articles. There was a strong increase in forest carbon-related papers beginning in 2009, which was notably later than avian group papers which began a strong increase in 2004. Over the past ten years, the average number of publications was 15.7 for the avian topic and 12.7 for the forest carbon component. The first authors of articles within our database were primarily based in the USA (United States of America) accounting for 34.9 percent (for avian) and 57.4 percent (for forest carbon) of the literature (Fig. 5). The most common study locations for both topics (i.e., where research was conducted, not shown on map) followed similar patterns, with most studies in the USA, followed by Australia, Canada, and China.

#### Forest metrics

**Fig. 6:**
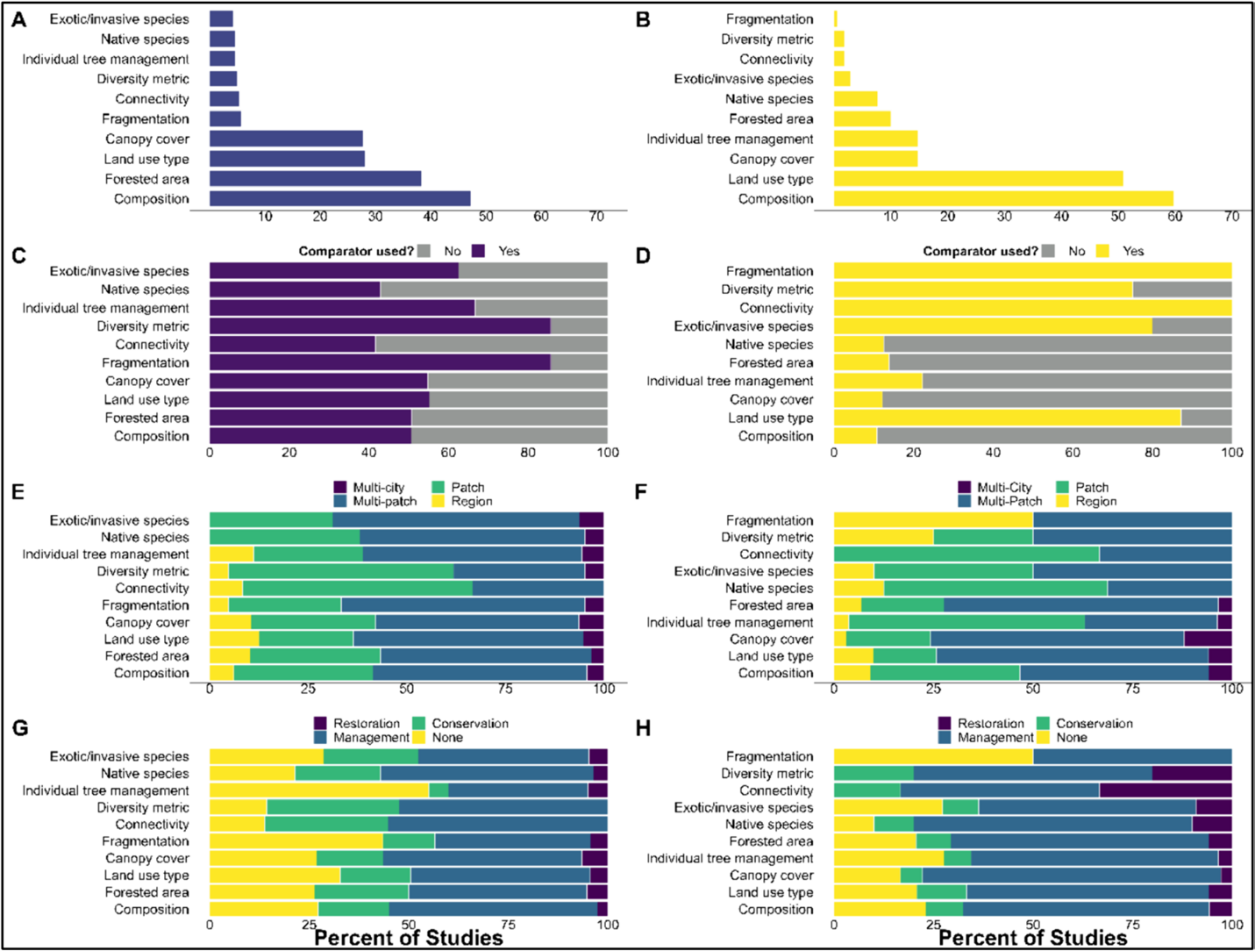
Comparison of avian and carbon groups for forest metrics. **A**,** B** The first row displays the percentage of articles by forest metric for each group. Rows** C**–**H** display proportional stacked bar charts for each forest metric. **E**,** F** Percent of articles within each forest metric, by comparator.** G**,** H** Percent of articles within each forest metric, by urban scale. (4) Percent of articles within each forest metric, by recommendation type

For all articles in the database, we coded the main forest metric measured (Table 3). The forest metric code represents the “intervention” (i.e., the change measured) which stands to affect either the “outcome” of avian success or carbon-based climate change mitigation. Of the forest metrics coded in both groups (avian and forest carbon), the overall dataset was highly dominated by articles including metrics of forest composition. Forest composition was considered in 101 forest carbon articles (60%) and 127 avian articles (46%). The avian group was dominated by the studies of forested area (104/277, 38%), land use type (78/277, 28%), and canopy cover (75/277, 27%). There were similarities in frequencies across the forest carbon topic which was dominated by the effects of land use type (86/169, 51%), individual tree management (25/169, 15%) and canopy cover (25/169, 15%) (Fig. 6). Connectivity, diversity, native species, exotic/invasive species and fragmentation metrics were least represented in both topics.

#### Avian metrics

**Fig. 7:**
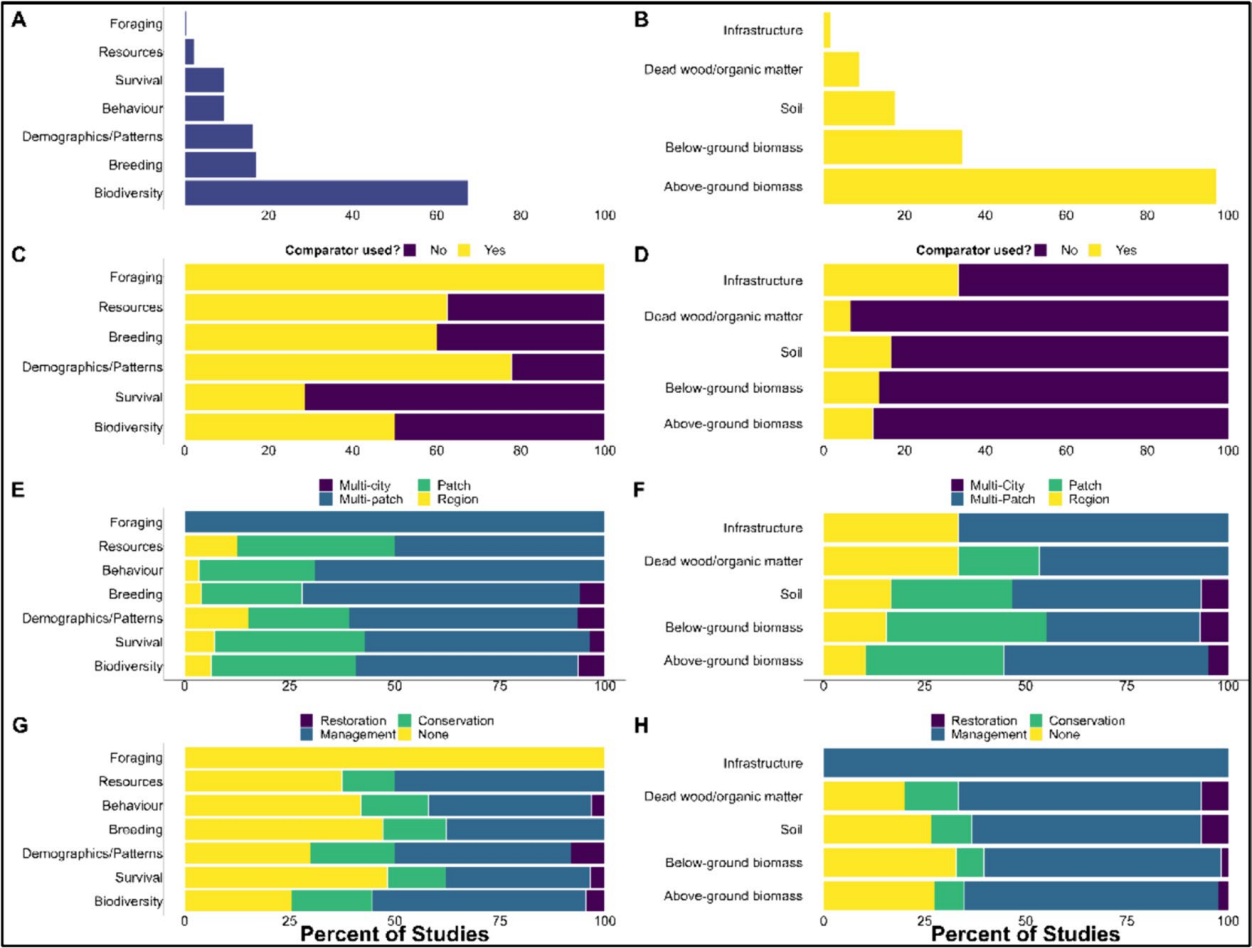
Comparison of avian metrics and carbon metrics measured for avian and carbon topic. **A**,** B** The first row displays the percentage of articles by forest metric for each group. Rows** C**–**H** display proportional stacked bar charts for each forest metric. (C, D) Percent of articles within each forest metric, by comparator. **E**,** F** Percent of articles within each forest metric, by urban scale. **G**,** H** Percent of articles within each forest metric, by recommendation type

For the avian group, avian success metrics extracted from articles were coded as “foraging,” “resources,” “behaviour,” “breeding,” “demographics/patterns,” “survival,” or “biodiversity”. If an article measured more than one metric, it was coded multiple times (e.g., “breeding” and “survival”). Most articles used metrics of biodiversity (e.g., abundance, richness, functional) (187/277, 68%). Metrics of breeding represented (46/277, 17%) closely followed by demographics/patterns (45/277, 16%) (Fig. 7). Metrics of behaviour (26/277, 9%), survival (26/277, 9%), resources (5/277, 2%) and foraging (1/277, 0.3%) all represent 10% or less.

#### Carbon metrics

For the forest carbon group, carbon metrics were coded as “aboveground biomass”, “belowground biomass”, “soil”, “organic/dead matter”, or “infrastructure”. If an article contained more than one measure of carbon it was coded multiple times (ex., “aboveground” and “belowground”). This group was heavily dominated by metrics of above-ground biomass to quantify carbon capture (165/169, 97%), followed by below-ground biomass (61/169, 36%) and soil (30/169, 18%) (Fig. 7). Metrics including dead and organic matter and infrastructural elements (timber, buildings) were heavily underrepresented in comparison to the dominant metrics (12/169, 7% and 3/169, 2%).

#### Urban scale

The urban scale considered was coded for all studies: “patch,” “multi-patch,” “region,” and “multi-city” (Table 3). Both topics had nearly identical patterns in the urban scale considered. The articles included in the map mainly assessed indicators at a multi-patch (bird: 150/277, 54% and forest carbon: 87/169, 51%) and patch (90/277, 32% and 56/169, 33%) scale, followed by region (23/277, 8% and 17/169, 10%). Both topics were least studied at a multi-city scale (14/277, 5% and 9/169, 5%).

## Recommendations

Recommendations were coded as either “management,” “conservation,” “restoration,” or “no recommendations.” If an article contained more than one type of recommendation, multiple responses were coded for that article (e.g., “management” and “conservation”). The avian component had a higher percentage of articles that contained no recommendations (100/277, 36%) compared to the carbon component (45/169, 27%).

Both topics were dominated by articles that provided recommendations related to management (144/277, 52% and 119/169, 70%). Generally, avian component papers contained more articles that provided conservation recommendations (58/277, 21%,) compared to the forest metric (19/169, 11%). Both topics were least represented by restoration-related recommendations (15/277, 5% and 11/169, 7%).

### Mapping the quality of studies relevant to the question

The most frequent study designs differed between forest carbon and avian articles. The forest carbon group was dominated by studies with no comparator (146/169, 86%). That is, a study may make comparisons within the study system (e.g., park vs. street land use), however, a control group was not integrated in the study design. The avian articles comparatively had just over half of the studies (156/277, 56%) that used a comparator (a control group was integrated into the study design).

The remaining articles did not use a comparator (119/277, 43%) and two articles did not provide enough information to determine if a comparator was used (2/277, 0.7%). When considering study timescale, the highest percentage of forest carbon studies were 0–1-year studies (59/169, 35%), followed by 2–5-year studies (31/169, 18%) (see Additional File 1). Studies longer than five years (6–10 or more than 10) accounted for 15 percent of studies (8/169, 5% and 13/169, 8% respectively). About a third of studies did not state a start or end year (58/169, 34%). The highest percentage of avian studies occurred over 2–5 years (127/277, 46%), followed by studies lasting 0–1 years (89/277, 32%). Studies longer than five years (6–10 or more than 10) accounted for the remaining 14 percent (18/277, 6% and 21/277, 8%). Finally, some studies did not state a start or end year (22/277, 8%).

### Knowledge gaps and clusters

**Fig. 8:**
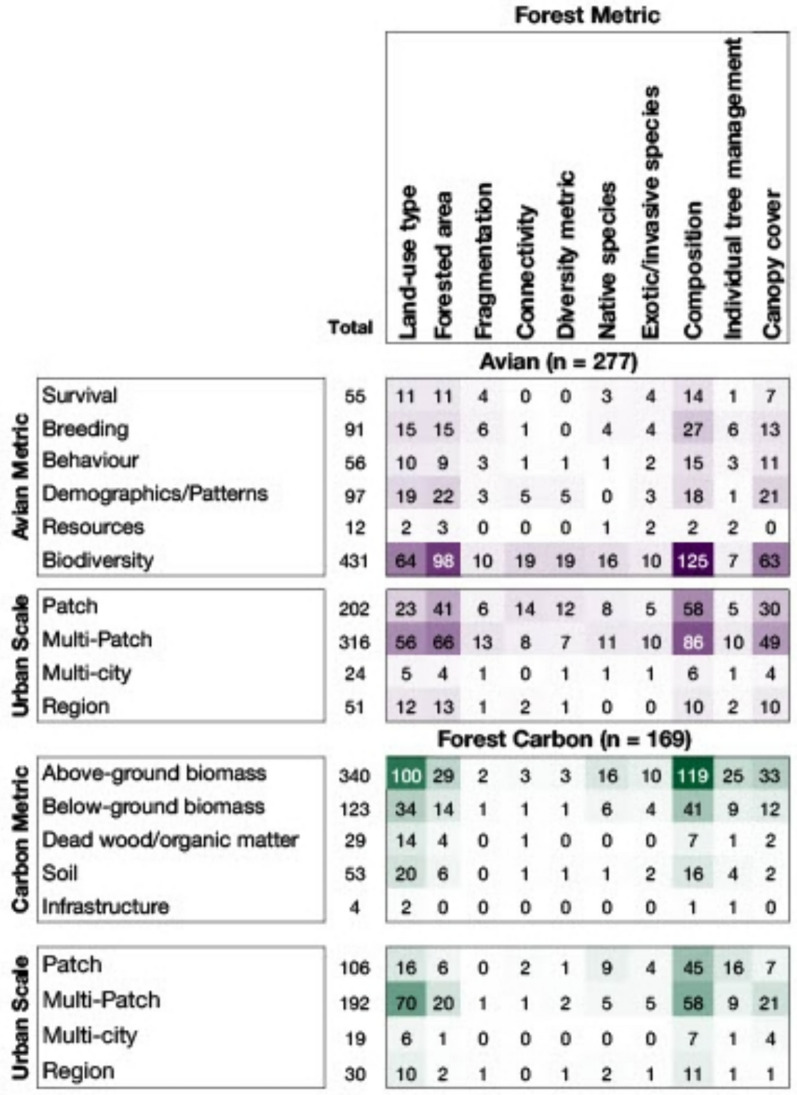
Knowledge cluster map visualising the number of articles for avian and forest carbon literature linked by forest management strategies (top) to avian topics, urban scale, and carbon metrics. Darker shades correspond to more studies containing data on both variables

We combined results across urban forest management interventions collected for both avian and forest carbon datasets (Fig. 8). We sorted the number of articles for each corresponding combination of indicators into bins (avian topics, carbon metrics, and urban scale), to visualise knowledge clusters and gaps. Our analysis confirms the knowledge clusters noted in the results for avian (in Fig. 8 namely, biodiversity) and carbon (in Fig. 8, above and belowground biomass) topics.

The most common forest management intervention and avian success combination was forest composition and biodiversity (125 articles), forested area and biodiversity (98 articles) and land-use type and biodiversity (64 articles). Metrics other than avian diversity such as survival, breeding, behaviour, demographics/patterns and resources were far less studied. Similarly, more fine-scale metrics of forest management like fragmentation, connectivity, diversity metrics, native species, exotic/invasive species, or individual tree management were highly understudied in combination with avian success (Fig. 8).

The most common forest management intervention and carbon metric combinations were above-ground biomass and forest composition (119 articles) and above-ground biomass and land-use type (100 articles). The least common forest management interventions across all carbon metrics were fragmentation, connectivity, diversity metric and native species (Fig. 8). Clear knowledge gaps also existed considering metrics other than above-ground and below-ground biomass. Although some articles considered soil, few included other carbon pools like dead wood/organic matter, or infrastructure.

Finally, both topics had knowledge clusters at the patch (avian 202, carbon 106) and multi-patch scale (avian 316, carbon 192). The avian topic had knowledge clusters for the combinations of composition and multi-patch (86), forested area and multi-patch (66) and composition and patch (58). Similarly, the carbon topic had knowledge clusters for composition and multi-patch (58), land use type and multi-patch (70) and composition and patch (45). Noticeable gaps for both topics were in studies conducted at the multi-city scale (24 avian and 19 carbon total).

## Limitations of the map

Coding, methodologies, exclusion parameters, benchmark papers, and team resources could all be considered limitations of our systematic map. Qualitative inquiry is necessarily subjective and shaped by the expertise of the research team. Though our approach of defining variables and codes for the avian and carbon topics were grounded in dominant themes in the literature, choices may differ if created by another research team with different expertise. For example, we chose to focus on urban forests as the system of interest, but other teams just as interdisciplinary as ours could have chosen terms such as “green space” or “green infrastructure,” which are used as metaphors with great definitional overlap to “urban forests” but relate to different bodies of literature [8].

We chose to exclude forest carbon literature related to tree plantations due to it being a system of management more relevant to rural contexts. However, we acknowledge there is likely relevant evidence base specific to forest management for carbon climate solutions within plantation literature that could potentially strengthen our current synthesis.

We included ten benchmark papers for each research topic, which is a conservative number given the breadth of literature we considered. Our search strategy and validation could have benefitted from a list of at least 20 articles for each topic to improve the robustness of our search and retrieved articles during the search process.

We also chose to combine our search string for theses in ProQuest by including keywords for both avian and carbon topics to streamline the search strategy and maximise our teams’ screening resources. The streamlined search string may have biased towards literature that combined our two topics (avian and forest carbon). We used “OR” rather than “AND” to minimise such bias and target each set of literature separately. Finally, our search was conducted solely in English and thus, our results were biased towards literature published in English. The depth of our evidence synthesis and geographic spread of areas studied would be improved had other languages been incorporated. Our ability to extract research outcomes and recommendations was also based on the clarity of reporting/writing within the articles included in our database.

## Conclusions

### Implications for research

Our systematic map highlights the potential for more integrated research on the dual outcomes of carbon-based climate mitigation and avian conservation within temperate urban forests, critical missed opportunities or knowledge gaps, and potential research challenges. While thus far, research on how urban nature-based solutions may provide climate mitigation or avian conservation (i.e., biodiversity conservation) has largely been siloed, there is both a great need and potential for this research to become more integrated. Currently, both avian and carbon research in cities is occurring at smaller urban spatial scales (patch, multi-patch), and lacking at larger spatial urban scales (multi-city, region). A major disconnect in current research is that field-based avian data (e.g., abundance, nest survival) and carbon data (e.g., aboveground biomass) that could be collected at the same spatiotemporal scales are often not integrated in the same study. Research on both avian conservation and carbon-based climate mitigation often make use of broad-scale readily available forest data, but regional or national efforts on carbon mapping or bird counts (e.g., Breeding Bird Atlases) are not coordinated in time and space, making large model integration of these dual outcomes of urban forest management difficult. Nonetheless, we show there is a substantial body of evidence to conduct an in-depth systematic review on avian diversity and aboveground biomass carbon metrics using broad forest management data (e.g., land use type and composition), which could deliver evidence-based recommendations for practice (Fig. 8). Our findings show there is opportunity for research and data collection efforts to be co-developed by urban foresters and conservation biologists to maximise both large-scale efforts and time-intensive fine-scale data collection to match data spatiotemporally [34].

Our findings highlight gaps in the inclusion of management recommendations in both carbon-based climate mitigation and avian conservation of urban forests scientific literature. Roughly 30% of articles in both topic databases did not include any conservation, restoration, or management recommendations for practitioners or land managers. Due to the highly applied outcomes of many of these studies, striving to include recommendations whenever appropriate can improve the translation of study outcomes from research to practice, particularly research that provides insights to the trade-offs and synergies of the multiple outcomes of urban nature-based solutions. The complex, multi-dimensional nature of the urban ecosystems means that recommendations from discrete research fields are limited in providing appropriate scientific advice [35], but this can be improved by using interdisciplinary and transdisciplinary approaches in research [36]. This integrated research is currently lacking, as evidenced by how strongly the two topics were siloed in very different journal outputs (Fig. 3).

There are clear knowledge clusters and gaps for urban forest management strategies for both avian conservation and carbon-based climate mitigation. A high number of articles were identified in our search (13,634 records without duplicates) with 3.3% (446) included in the systematic map. We expect this lower number of mapped articles is linked to the frequent use of keywords related to nature-based solutions and conservation which cover subjects unrelated to our research questions. Overall, our map will allow researchers to fill existing gaps in the literature through new research investigations, meta-analyses, or systematic reviews. Researchers may look to areas scored 0–25 (in white and lighter shades) in the knowledge cluster analysis (Fig. 8) to inspire novel and underexplored approaches. This would include prioritising research at larger urban spatial scales (multi-city, region), understanding the outcomes of the least understood interventions (e.g., fragmentation, connectivity, diversity, native species), and the least quantified avian metrics (resources, behaviour, and survival). Focusing efforts on these identified knowledge gaps would benefit both the scientific community and practitioners working on urban nature-based solutions.

### Implications for policy/management

The findings of our systematic map indicate patterns of strong evidence as well as understudied parameters in the current state of knowledge of urban nature-based solutions and the implications for policy/management. Our synthesis shows there is a substantial body of evidence relevant to informing policy related to broad urban forest management strategies (e.g., land use type, composition) at local urban scales (e.g., patch, multi-patch). This body of evidence has been increasing consistently over the last decade. Policymakers influencing urban forest management should take note of the differences in the bodies of evidence related to climate mitigation and biodiversity. In particular, this map shows the depth of evidence for forest management intervention at different scales and where particular recommendations according to those interventions can be found. Most existing knowledge on bird conservation (namely biodiversity) is related to forested areas, composition, canopy cover and land use type, as opposed to forest connectivity, diversity, or the presence of native tree and shrub species (Fig. 8). The carbon knowledge base similarly focused on broad-scale forest management metrics like composition and land-use type to estimate above and belowground biomass. However, the body of evidence for both avian and carbon outcomes present a scale-mismatch between knowledge of forest management strategy (e.g., individual tree management) and scale of application (patch). For example, the majority of studies considered forest metrics at broad scales like land use type, or composition, yet were conducted at a patch or multi-patch scale. Our impression is that this is likely a result of the scale of readily accessible datasets containing forest data (land use, canopy, composition classification).

While the spatial scale of research (patch or multi-patch) is one of interest to practitioners and land-managers, the focus on broad-scale forest management strategies leads to a spatial mismatch between the scale of research and the scale relevant for conservation practice of management action [37]. Management needs at the patch scale often require finer scale information to be relevant to managers (e.g., what to plant, where to plant it) rather than land use classification or overall canopy cover. The scale of governance and the scale of ecological processes are often highly mismatched in urban systems [38].

Biodiversity, as an outcome of avian conservation, vastly outnumbered the five other outcomes combined in our systematic map. We speculate that this is similarly due to data available as with the forestry data, as avian metrics were most often measured in broad diversity indices (e.g. abundance, richness) which rely on readily available datasets like eBird rather than localised and time-intensive field data. Even when local field data collection takes place, methods such as point counts are far easier to perform than more detailed, intensive studies. In addition, biodiversity research in general has exploded since the 1990s [39], and is often the focus of media attention and funding calls.sec

Yet there are critical challenges for policy and management action to be guided by a broad metric such as biodiversity, despite its undeniable ecological importance. Managing ‘biodiversity’ as a practitioner is vague and makes actionable goals or specific best practices difficult to articulate and may decrease the capacity to integrate with carbon-based climate change mitigation, which offers simpler targets to set. While policy and programming may touch on biodiversity as a concept, there is often a lack of clear legislation for biodiversity conservation as a whole. In Canada for example, 201 laws consider biodiversity, but there are no legislative imperatives to conserve and protect biodiversity at a systematic level [40]. Instead, conservation laws such as the Canada Wildlife Act, the Species at Risk Act, and the Migratory Birds Convention Act primarily function and focus on protected areas or are species-centric, with a distinct weakness when it comes to considering biodiversity or ecosystems generally [41]. Similarly, the scales at which carbon is, or may be, regulated or managed ranges from the municipal or institutional level, through Climate Action Plans, to national levels. Clear gaps in knowledge related to tree diversity’s contribution to carbon climate solutions further amplify these mismatches. As laws are the drivers of governance and program structures that guide practitioners, there is a disconnect between the metrics for which we have the most current knowledge (i.e., biodiversity) and those metrics that are written in policy (i.e., % of protected area, presence of listed species-at-risk).

## Supplementary Information

Supplementary Material 1.

Supplementary Material 2.

Supplementary Material 3.

Supplementary Material 4.

## Data Availability

All data materials and code are publicly available and stored on GitHub: https://github.com/Kayleighht/Syst_Map_Avian_Forest
