# Supplementary material for: Existing evidence on the effect of urban forest management in carbon solutions and avian conservation: a systematic literature map
Source: Environ Evid. 2024 Oct 3;13:23. doi: 10.1186/s13750-024-00344-3 (PMC11448182; doi:10.1186/s13750-024-00344-3)
Supplement: Supplementary file 1 — Supplementary Material 1. [file 13750_2024_344_MOESM1_ESM.docx]

**Additional File 1**

**Table of Contents:**

1. **Table. 1:** List of specialist websites searched
2. **Screening Questions:** Decision tree questions for title and abstract screening
3. **Call for Evidence:** Open call for evidence to collect forms of grey literature
4. **Figure 1.** Knowledge cluster displaying the number of articles in the sub-categories of avian metrics
5. **Figure 2.** Bar chart displaying the number of indicators measured for avian topic and carbon topic literature.
6. **Figure 3.** Timescale considered in each study, by percent of total studies for avian and forest carbon group literature, according to four timescales bins: 0-1 years, 2-5 years, 6-10 years or >10 years. Note, this indicator captures the total timescale considered and does not consider gaps in sampling years.
7. **Table 2.** Description of consistency checks conducted at title and abstract and full-text screening phases between a minimum of two reviewers.

**Table 1.** List of specialist websites to be searched for each respective topic.

| **Carbon** | **Avian and Species Risk** |
| --- | --- |
| 1. Tree Canada  2. Canadian Institute for Climate Choices  3. Nature Canada  4. Forests Ontario  5. Nature Conservancy Canada  6. David Suzuki Foundation  7. World Wildlife Fund Canada  8. Forest Stewardship Council  9. Arbor Day Foundation  10. National Aboriginal Forestry Association | 1. Ducks Unlimited  2. Birds Canada  3. National Audubon Society  4. Partners in Flight  5. American Ornithological Society  6. American Bird Conservancy  7. Bird Life International  8. Nature Canada  9. Cornell Lab for Ornithology: Celebrate Urban Birds  10. Smithsonian Institute: Migratory Bird Center |

**Screening Questions- Title and Abstract**

Urban Forest- Avian/Species at Risk Conservation

**Aim:** To identify the main themes in urban ecological research that have addressed urban forest management strategies for avian and/or species at risk conservation and protection.

If there is one “No” to the questions below, the study is to be excluded.

If there is any doubt or questions, the study is to be included.

**1. Population: Is the population in the North (23.5° N to 66.5° N) or South (23.5° S to 66.5° S) temperate regions?** Yes/No

**2. Population: Is the study conducted in an urban area?** Yes/No

If it is unclear whether the area is urban, we will use the UN Statistical Commissions international threshold to include cities, towns, and semi-dense areas with a population of at least 5,000 inhabitants and a density of at least 300 inhabitants per km². [(UN Statistical Commission 2020)](https://paperpile.com/c/ceZXYU/haN6). Tend towards inclusion for full text screening if still uncertain.

**3. Population:** **Is this a population of avian species or species at risk?** Yes/No

Bird species can be residents (e.g., non-migratory) or migratory at any life stage. Only studies located in urban ecosystems are considered.  Exotic species and non-natives that have been naturalized to North America will be included (e.g., European starling). Species at risk should be specific to Canada as noted in the Federal Government's registry: <https://laws.justice.gc.ca/eng/acts/s-15.3/page-10.html>.

**4. Does the article evaluate the effect of urban forest management strategies as it relates to avian species and/or species at risk?** Yes/No

Urban forest management strategies include habitat protection, tree planting, composition, structure, reforestation, diversity, maintenance, canopy cover etc.

Look for words like nesting, nest sites, nesting habitat etc. which all could include components of the urban forest.

**5. Is this a review article?** Yes/No

Review papers and policy discussions are not eligible papers and will be excluded. However, all relevant review articles are “Exclude (relevant review)”. Each review will be examined, and the reference list will be examined for “relevant articles to be included in the dataset. All policy discussions should be binned as “Exclude (Policy)”.

**Screening Questions- Title and Abstract**

Urban Forest- Carbon Climate Solutions

**Aim:** To identify the main themes in urban ecological research that have addressed urban forest management for climate regulation (e.g., carbon storage and sequestration)

If there is one “No” to the questions below, the study is to be excluded.

If there is any doubt or questions, the study is to be included.

**1. Population: Is the population in the North (23.5° N to 66.5° N) or South (23.5° S to 66.5° S) temperate regions?** Yes/No

**2. Population: Is the study conducted in an urban area?** Yes/No

If it is unclear whether the area is urban, we will use the UN Statistical Commissions international threshold to include cities, towns, and semi-dense areas with a population of at least 5,000 inhabitants and a density of at least 300 inhabitants per km². [(UN Statistical Commission 2020)](https://paperpile.com/c/ceZXYU/haN6)

**3. Population:** **Is this a population of trees and/or woody species?** Yes/No

Any species with a minimum diameter at breast height (DBH) of 5 cm² [(Nowak *et al.* 2008)](https://paperpile.com/c/ceZXYU/dlhY). For text that mentions trees, woody species, shrubs, or urban forest vegetation, tend towards inclusion.

**4. Does the article discuss climate solutions (e.g., carbon sequestration and/or carbon storage)?** Yes/No

This can be defined in several ways, including, carbon assimilation, concentrations of carbon, carbon emissions and or CO2 concentration reductions, carbon stocks and if unclear, tend towards inclusion.

**5. Is this a review article?** Yes/No

Review papers and policy discussions are not eligible papers and will be excluded. However, all review articles are “Exclude (relevant review)”. Each review will be examined, and the reference list will be examined for “relevant articles to be included in the dataset. All policy discussions should be binned as “Exclude (Policy)”.


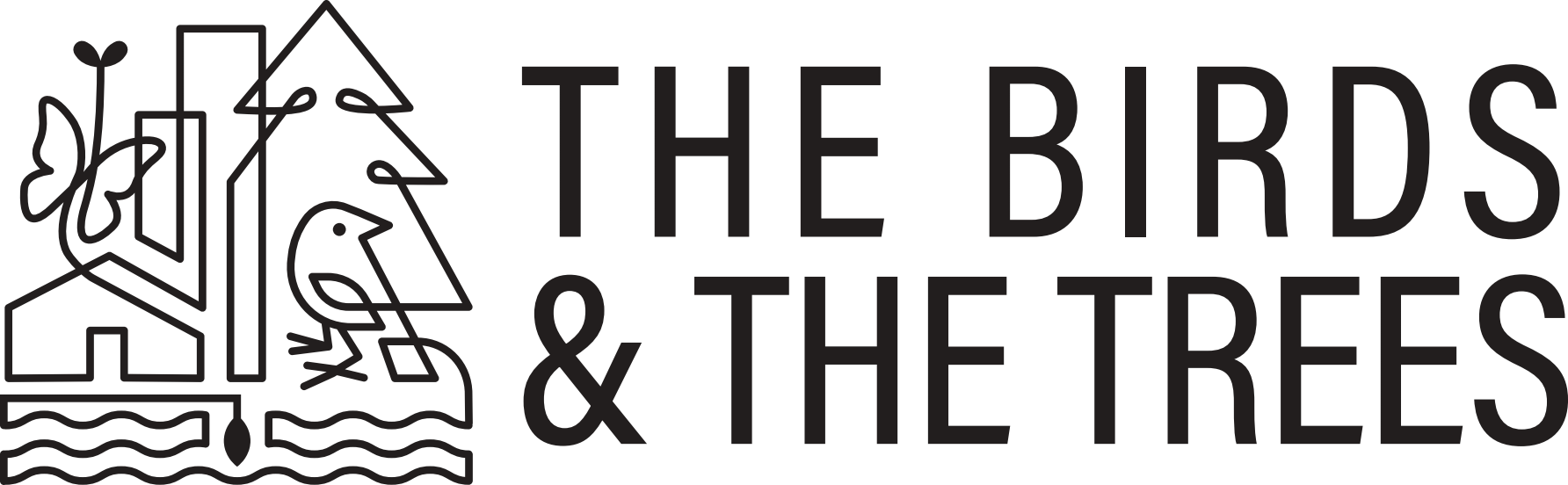


**Evidence Call for Grey Literature**

The Ziter Urban Landscape Ecology (ZULE) lab (<https://www.carlyziter.com>) at Concordia University, in collaboration with Environment and Climate Change Canada (ECCC), needs your help with an ongoing systematic map evidence search:

**“What evidence exists for the use of urban forest management in nature-based carbon solutions and bird conservation. A systematic map protocol.”**

**Aim:**

The primary goal of this review is to synthesize and compile evidence related to best practices in urban tree management to support climate change mitigation as well as birds and other species at risk. We will be searching two bodies of literature, (1) urban forest management interventions for climate mitigation, namely carbon storage and sequestration and, (2) effective conservation efforts for urban avian and species at risk.

While the focus of our review is to primarily inform Canadian urban forest management, studies from all temperate locations in the world are also eligible for inclusion in this review.

**What are we looking for:**

The review team is sourcing studies and literature on this topic in the form of:

- Academic research and theses
- Reports (government, NGO, consultants, community)
- Conference papers
- Workshop summaries
- White papers
- All other literature “that is produced on all levels of government, academics, business, and industry in print and electronic formats, but which is not controlled by commercial publishers” (*4th International Conference on Grey* *Literature, 1999*)

**
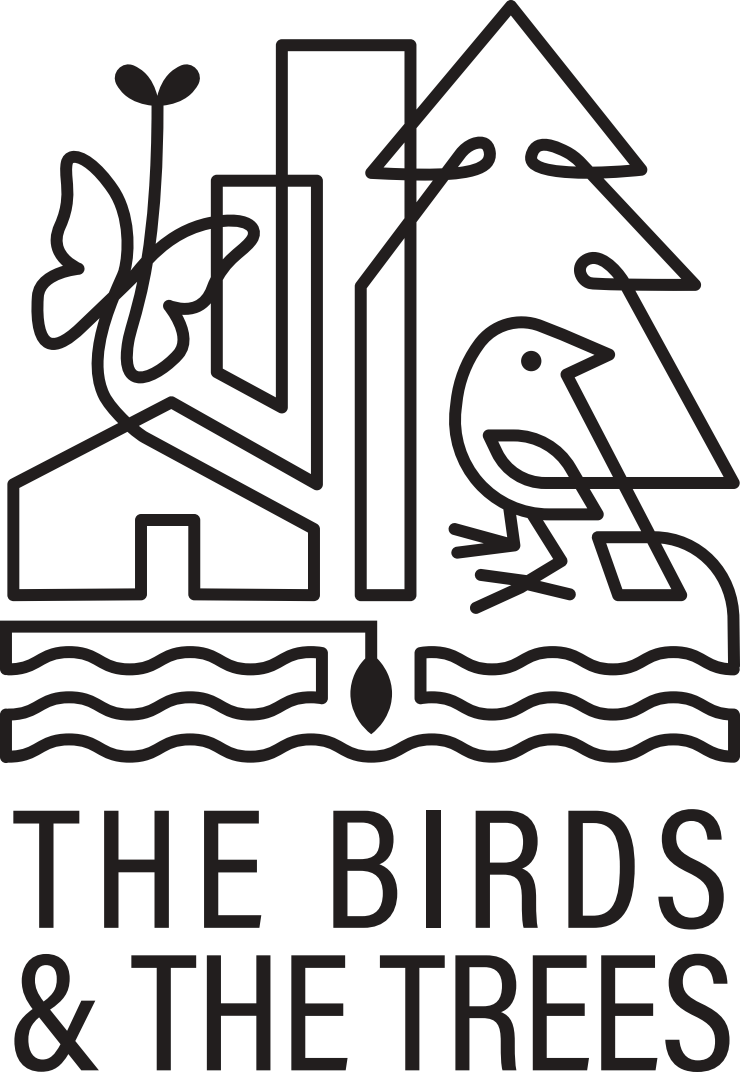
**

**Get in touch:**

If you can provide any grey literature (or relevant published material) on this subject, please send any information to [kayleigh.hutttaylor@concordia.ca](mailto:kayleigh.hutttaylor@concordia.ca) by November 18, 2022.

**Thank you!**

*This call for evidence was adapted from the Canadian Centre for Evidence-Based Conservation (CEBCEM) at Carleton University

**
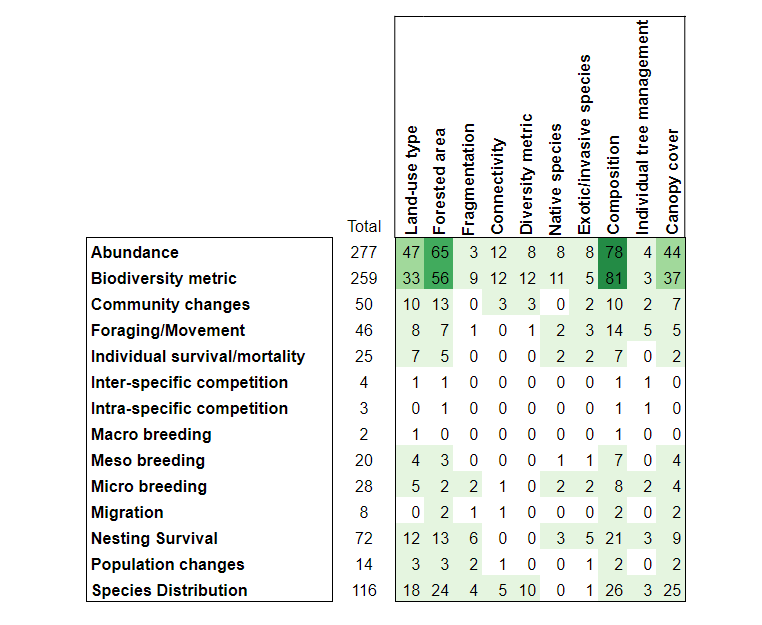
**

**Figure 1.** Knowledge cluster displaying the number of articles in the sub-categories of avian group (left column) related to urban forest management categories (top).


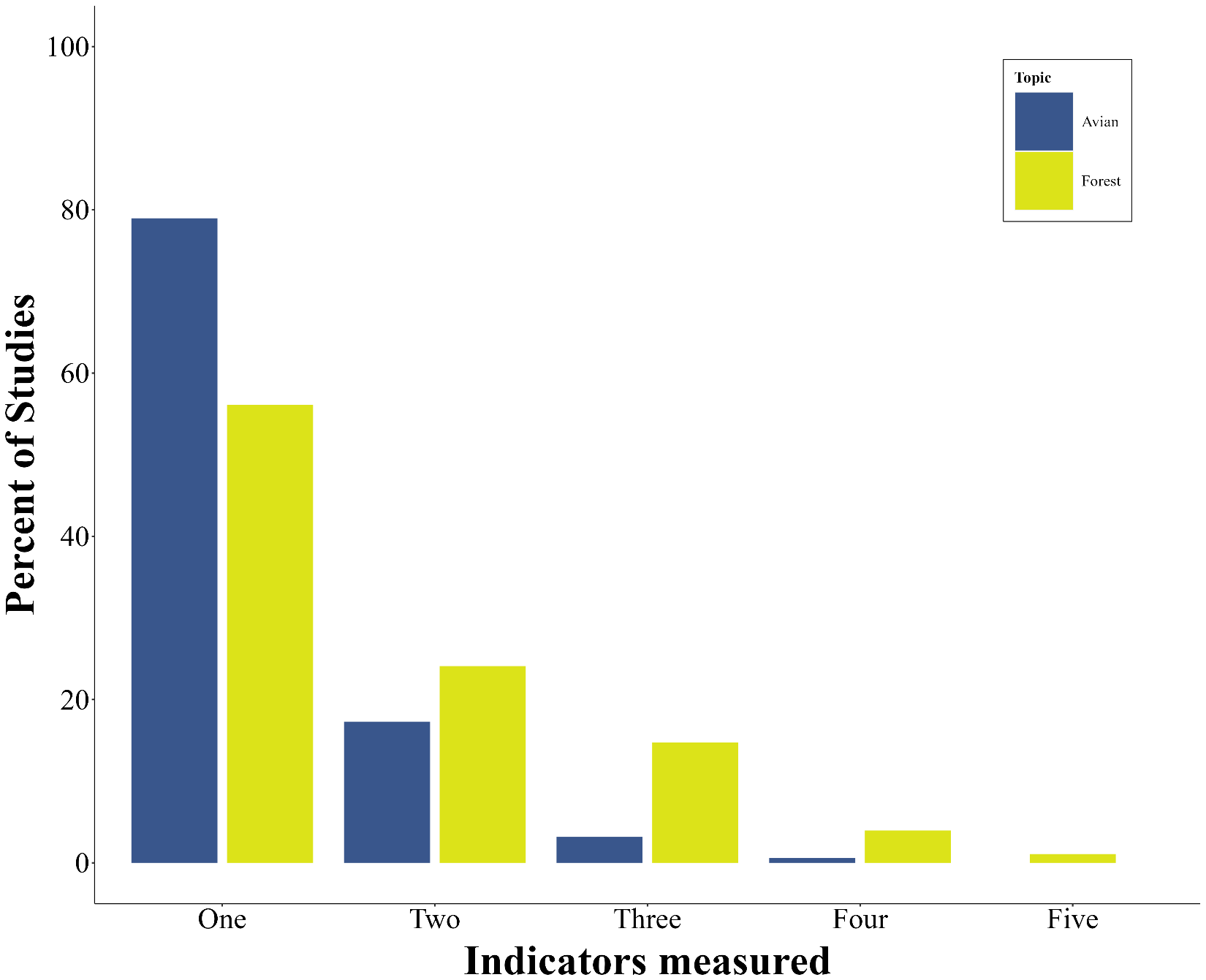


**Figure 2.** Bar chart displaying the number of indicators measured for avian topic and carbon topic literature.


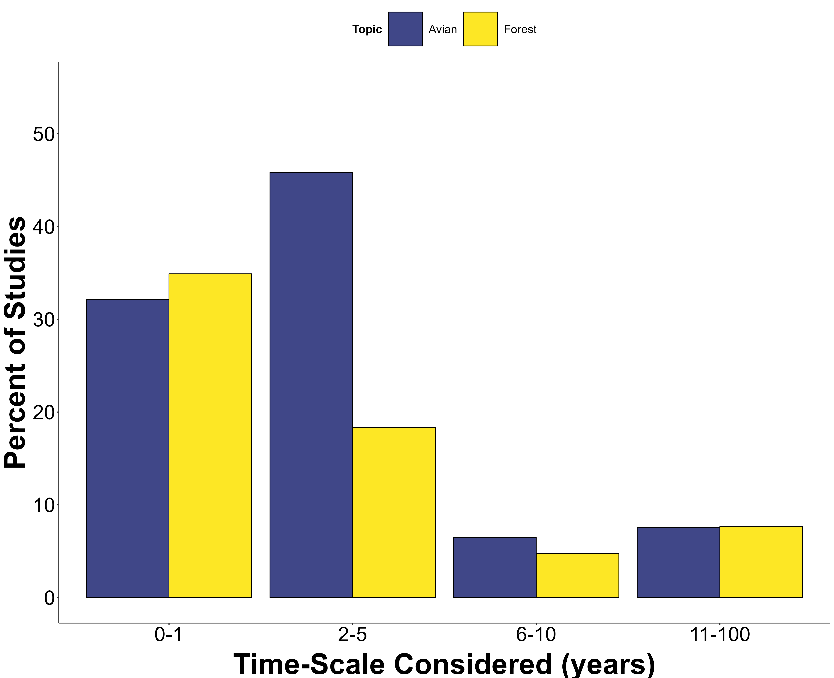


**Figure 7**. Timescale considered in each study, by percent of total studies for avian and forest carbon group literature, according to four timescales bins: 0-1 years, 2-5 years, 6-10 years or >10 years. Note, this indicator captures the total timescale considered and does not consider gaps in sampling years.

**Table 2.** Description of consistency checks conducted at title and abstract and full-text screening phases between a minimum of two reviewers.

| **Title and Abstract**  ***Avian Forest Carbon*** | | **Full Text**  ***Avian Forest Carbon*** | |
| --- | --- | --- | --- |
| 200/7,857 | 150/5,929 | 10/1,649 | 10/1,264 |
